# Pyramiding, alternating or mixing: comparative performances of deployment strategies of nematode resistance genes to promote plant resistance efficiency and durability

**DOI:** 10.1186/1471-2229-14-53

**Published:** 2014-02-22

**Authors:** Caroline Djian-Caporalino, Alain Palloix, Ariane Fazari, Nathalie Marteu, Arnaud Barbary, Pierre Abad, Anne-Marie Sage-Palloix, Thierry Mateille, Sabine Risso, Roger Lanza, Catherine Taussig, Philippe Castagnone-Sereno

**Affiliations:** 1 INRA, UMR1355 INRA/UNSA/CNRS, Institut Sophia Agrobiotech, BP167, Sophia Antipolis F-06903, France; 2 INRA, UR1052, Génétique et Amélioration des Fruits et Légumes, CS 60094, Montfavet, Cedex F-84143, France; 3 IRD, UMR CBGP, Campus de Baillarguet, CS30016, Montferrier-sur-Lez, Cedex F-34988, France; 4 Chambre d’Agriculture des Alpes Maritimes, MIN Fleurs 17 - Box 85, Nice, Cedex 06286, France; 5 APREL, Association Provençale de Recherche et d’Expérimentation Légumière, Route de Mollégès, Saint-Rémy de Provence F-13210, France

**Keywords:** Breeding strategy, *Capsicum* spp, *Meloidogyne* spp, Resistance gene deployment, Root-knot nematodes, Sustainable crop protection, Virulence emergence

## Abstract

**Background:**

Resistant cultivars are key elements for pathogen control and pesticide reduction, but their repeated use may lead to the emergence of virulent pathogen populations, able to overcome the resistance. Increased research efforts, mainly based on theoretical studies, explore spatio-temporal deployment strategies of resistance genes in order to maximize their durability. We evaluated experimentally three of these strategies to control root-knot nematodes: cultivar mixtures, alternating and pyramiding resistance genes, under controlled and field conditions over a 3-years period, assessing the efficiency and the durability of resistance in a protected crop rotation system with pepper as summer crop and lettuce as winter crop.

**Results:**

The choice of the resistance gene and the genetic background in which it is introgressed, affected the frequency of resistance breakdown. The pyramiding of two different resistance genes in one genotype suppressed the emergence of virulent isolates. Alternating different resistance genes in rotation was also efficient to decrease virulent populations in fields due to the specificity of the virulence and the trapping effect of resistant plants. Mixing resistant cultivars together appeared as a less efficient strategy to control nematodes.

**Conclusions:**

This work provides experimental evidence that, in a cropping system with seasonal sequences of vegetable species, pyramiding or alternating resistance genes benefit yields in the long-term by increasing the durability of resistant cultivars and improving the long-term control of a soil-borne pest. To our knowledge, this result is the first one obtained for a plant-nematode interaction, which helps demonstrate the general applicability of such strategies for breeding and sustainable management of resistant cultivars against pathogens.

## Background

Crop production in open-field and greenhouse conditions is strongly committed to the intensification of agriculture and reduction of pesticide use, principally in response to regulatory, health, environmental and commercial constraints. Pest and disease resistant cultivars are key elements for pathogen control, and the development of effective rotation systems [1]. Therefore, breeding for disease resistance has become an important topic in most crop improvement programmes. Many resistance genes (*R*-genes) are already being used in agriculture and face three major constraints: (1) the limited number of cultivated species with *R*-genes available; (2) the lengthy process of breeding *R*-varieties with high agronomic standard; (3) the emergence of virulent populations, able to overcome the resistance conferred by some of the *R*-genes, which is favoured by the continuous monoculture of cultivars carrying the same *R*-genes (for review, see [2] for fungal pathogens, [3] for viruses, [4] for insects, and [5] for nematodes). The promotion of durable resistance, defined as a resistance remaining effective for a long period of time during its widespread cultivation in environments favouring disease development [6,7], is still an ongoing quest and a major issue.

Recent research showed that management strategies of the resistance sources can increase the durability of the resistance. Firstly, the choice of the *R*-gene and of the genetic background in which the major *R*-gene is introgressed are determinant and can be optimized [8,9]. As an alternative to single-gene deployment, combinations of several alleles in a single genotype, also called gene pyramiding, are expected to increase the number of mutations required from the pathogen for virulence, which may also increase the associated fitness penalty [10-12]. Gene pyramiding has been successfully applied in combining multiple genes for qualitative disease resistance such as bacterial blight resistance [13] and blast resistance [14] in rice, powdery mildew resistance in wheat [12]. The durability of the resistance depends on the time taken for new mutations or recombinations to generate the matching combination of virulence factors in the pathogen population and for that pathotype to establish itself in the population. However, the requirement for agronomic performance, does not always allow the breeder to fully use the genetic diversity available in *R*-genes and genetic backgrounds. Therefore, increased research efforts have been made to identify spatio-temporal *R*-cultivars deployment strategies and cultivation methods to maximize durability [15-19]. The sequential (or alternating) use of distinct *R*-genes in rotation (if a specificity of virulence has been shown) or the mixture of lines carrying distinct *R*-gene(s) in the same plot, may lower the emergence of virulent populations by diversifying the selection pressures for mutations in avirulence genes and avoid the potential bust of resistance when a single *R*-gene is deployed over a large area [20-22]. Pyramiding, mixture of lines, and alternation deployment have not been widely used, owing to the time required for breeding assortments of *R-*genes into elite cultivars and the difficulty to implement field experiments in practice. Therefore, in the literature, building new sustainable cropping systems relies on mathematical models of pathogen evolution [15,23-26]. The gene pyramiding strategy was empirically used in several crops. However, the pyramiding, mixture and alternating strategies were never compared experimentally, especially for intensive vegetable cropping systems. These strategies were recently compared in a review of theoretical and experimental use of pesticides in agriculture and drugs in medicine [27]. It showed that all the strategies can succeed in delaying the evolution of pathogen populations towards resistance to the pesticides or drugs, depending on the genetics of drugs resistances, but the combination of molecules (equivalent to the pyramiding strategy) outcompetes the other strategies.

Plant-parasitic nematodes are among the most damaging and uncontrollable pests of cultivated crops causing severe economic losses in world agriculture, estimated to $US 121 billion per year [28]. The specialized and intensive vegetable crops agriculture is becoming particularly vulnerable to a few species belonging to the group of root-knot nematodes (RKNs, *Meloidogyne* spp.). They are obligate plant endoparasites, found throughout the world in tropical, subtropical and warm-temperate areas in which several nematode generations can be completed per year. These polyphagous nematodes are one of the main pathogens on many Solanaceous crops but also of most vegetable crops (cucurbits, lettuce) which are part of the rotation in intensive protected cropping systems [29]. The parasite pressure due to these soilborne pests in vegetable crops has increased steadily following the new regulations that have withdrawn the use of most chemical nematicides [30,31]. Host resistance is considered as an important component of integrated management of RKNs [32]. Because few *R*-genes acting against these pests are currently available, it is urgently needed to protect them and promote their durability.

In pepper, five distinct *Me* genes were identified in local populations, that control different species of *Meloidogyne* (*M. arenaria, M. incognita, M. javanica, M. hapla*) [33-35]. Some of these genes have only recently been used in plant breeding and the risk of resistance breakdown by pathogen adaptation has already been demonstrated under laboratory experiments with high inoculum pressure of nematodes [36,37]. Previous experimental studies showed that two genes (named *Me1* and *Me3*) differ in their mode of action, particularly in the spatio-temporal localisation of the hypersensitive reaction (HR) triggered by RKN penetration into the roots [37,38]. *Me3* induces early cellular necrosis in the root epidermis adjacent to the juveniles whereas *Me1* induces a late hypersensitive reaction in the vascular cylinder of infected roots, thus inhibiting the development of egg-laying females [39,40]. Interestingly, virulent populations were obtained for *Me3*, both in natural (i.e., in the field) and artificial (i.e., in the laboratory) conditions, whereas, to date, no evidence showed the emergence of *Me1*-virulent populations [36,37], which suggests a possible relationship between the mode of action of these *R*-genes and their durability. In this pathosystem, virulence gain was shown to be highly specific to the targeted *R*-gene and associated to a reproductive fitness cost [37]. Such trade-offs between virulence traits and fitness-related traits suggest that, although the resistance can be broken, it may be preserved by pertinent management strategies.

The interest in the RKN model system is based on its originality compared to other plant pathogens. First, the parasitic pressure that is applied by RKNs to their host plants is theoretically low: small population size (a few hundred juveniles in one egg-mass (EM) as the total progeny of a female), long biological cycle (about eight weeks at 20°C). Second, the biological features of RKNs that govern their evolutionary potential should not favour the emergence of virulent populations, according to McDonald & Linde [2]: lack of sexual reproduction for the major species (obligatory mitotic parthenogenesis), active dispersal capacities reduced in soil. However, as observed in other pathosystems, previous studies in tomato and pepper showed that virulence can emerge in laboratory as well as in fields, depending on the resistance gene used, and the genetic background it has been introgressed in [36-38]. The RKN model studied here will contribute to the generalization of strategies for the breeding and deployment of resistant cultivars.

In this study, we evaluated several *R*-gene deployment strategies to implement a rational use of pepper *R*-cultivars, with the objective to improve the sustainable management of RKNs in vegetable cropping system. Experiments were conducted in climate-controlled rooms and in greenhouses, under 3-year field conditions, comparing i) the use of a single *R*-gene introgressed in a partially resistant vs. a susceptible genetic background, ii) the alternance of two *R*-genes in rotation, iii) the mixture of genotypes bearing distinct *R*-genes in the same plot, and iv) the pyramiding of two *R*-genes in a single genotype.

## Results

The five pepper (*Capsicum annuum*) genotypes used in this work are inbred lines with differential resistances to RKNs. Doux Long des Landes (DLL) is a susceptible cultivar. The two resistant doubled-haploid (DH) lines, DH149 and DH330 produced through in vitro androgenesis were previously described [33]; they are homozygous for the *Me3* and *Me1* genes, respectively [41]. Two F1 hybrid were also used, one carrying *Me1* in its heterozygous state in the DLL susceptible genetic background (F1 [DH330 × DLL]), and one combining the two *R-*genes *Me3* and *Me1* (F1 [DH149 × DH330]).

### Climate controlled room experiment

**Figure 1:**
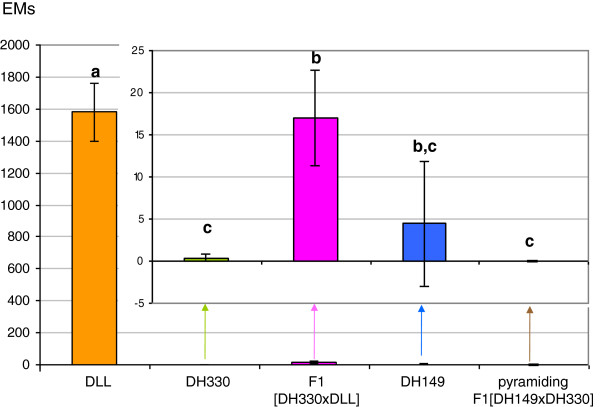
**Comparison of mean number of egg-masses (EMs) on peppers maintained in climate controlled room after inoculation with 5000 avirulent *M. incognita *juveniles (mean of 70 to 100 replicates ± standard error).** DLL was the susceptible cultivar as control, DH149 is homozygous resistant *Me3/Me3*, DH330 is homozygous resistant *Me1/Me1*. Modalities with different letters display significant differences at P = 0.05.

Three *M. incognita* isolates were used: *M. incognita* Morelos is a *Me1*- and *Me3*-avirulent isolate; the *Me3*-virulent laboratory-selected isolate was obtained from *M. incognita* Morelos and reared by successive re-inoculation on the *Me3*-pepper DH149 line as described in Material and methods; the *Me3*-virulent natural isolate was collected on resistant peppers carrying *Me3* in the experimental field of CREAT, La Baronne, France. Five hundred to 5000 hatched second-stage juveniles (J2s) were inoculated to six to seven-week-old plants according to the experiment described in Material and method. Comparison of the pepper genotypes for the number of egg-masses (EMs) after a drastic pressure of inoculum (5000 *M. incognita* juveniles per plant) shows that all *R-*peppers were not at all or very slightly infected (EMs from 0 to 18.4 ± 6.5) compared to the susceptible control DLL (1579.7 ± 184.3) (χ^2^ = 28 to 57, ddl = 4, p < 10–3) (Figure 1). The *Me1 R*-peppers with fifty percent of susceptible DLL genetic background (F1 [DH330 × DLL]) had mean EMs of 17.0 ± 5.6 compared to 0.4 ± 0.4 for DH330. A total of 35 and 1192 EMs were collected from the roots of 97 DH330 and 70 F1 [DH330 × DLL] *R*-peppers, respectively. No progeny could be obtained from EMs collected on DH330 by repetitive inoculation. The reproductive rate RR = 0 ( i.e., the number of eggs produced by one female, see Method). A first generation progeny was obtained from EMs collected from the F1 [DH330 × DLL] but did not succeed in producing a second generation so that RR was not determined. A total of 479 EMs were collected from the roots of 108 DH149 (*Me3*) *R*-peppers. These EMs contained 264 ± 23.5 eggs per EM (data not shown). A virulent line was reared by successive re-inoculation on DH149 *Me3*-peppers. After three successive re-inoculations, the mean number of eggs per EM (RR) was 897.9 ± 62.0 (14 replicates). No EM was obtained from the F1 [DH149 × DH330] *R*-peppers combining *Me1* and *Me3.*

**Figure 2:**
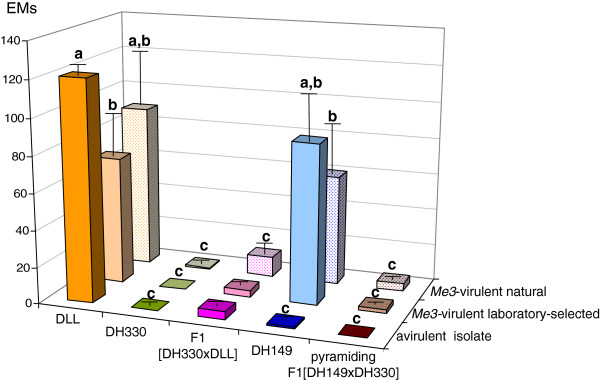
**Comparison of mean number of egg-masses (EMs) on peppers maintained in climate controlled room after inoculation with 500 juveniles of avirulent or *Me3*-virulent laboratory-selected or naturally *Me3*-virulent *M. incognita *isolates (mean of 10 to 20 replicates ± standard error).** DLL was the susceptible cultivar as control, DH149 is homozygous resistant *Me3/Me3*, DH330 is homozygous resistant *Me1/Me1*. Modalities with different letters display significant differences at P = 0.05.

Figure 2 compares the reproduction potential of avirulent and *Me3*-virulent laboratory-selected or natural *M. incognita* isolates on the different pepper genotypes after inoculation with 500 juveniles (10 to 20 replicates). All three isolates succeeded in producing EMs on the susceptible pepper DLL, mean of EMs ranging from 69.6 ± 17.3 for the virulent laboratory-selected isolate to 89.5 ± 28.7 for the natural virulent isolate. The avirulent isolate was mostly unable to reproduce on the resistant peppers (EM values ranging from 0 to 4.4 ± 2.0) except on *Me1 R*-peppers with fifty percent of susceptible DLL genetic background (F1 [DH330 × DLL]) for which 65 EMs were obtained from 15 F1 plants. The two *Me3*-virulent isolates were able to reproduce on *Me3*-peppers (mean EMs = 87.2 ± 26.6 and 60.3 ± 24.8 for virulent laboratory-selected and natural isolates, respectively), but they were not able to infect any *Me1*-pepper tested (DH330, F1 [DH330 × DLL], and F1 [DH149 × DH330]); 11 ± 7,1 EMs were counted on F1 [DH330 × DLL] infested with the *Me3-*virulent natural isolate but only 59 eggs were obtained from the EMs and they were not viable (data not shown).

### Field experiment

**Figure 3:**
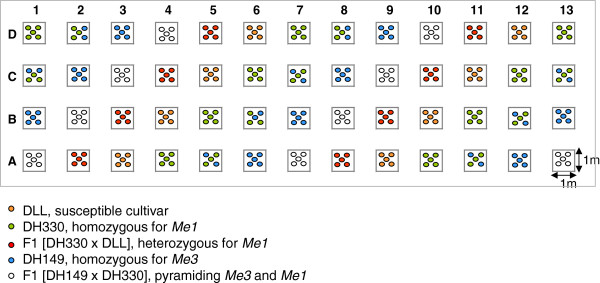
**Plan of the 3-years field experiment in the plastic tunnel.** Six cultivation modalities were compared during three successive years: 1) the succession of the same *R-*gene (*Me1*), when introgressed in a partially resistant genetic background (DH330), 2) the succession of the same *R-*gene (*Me1*) in a F1 hybrid issued from a cross with a susceptible genotype [DH330 x DLL]), 3) the alternance of single *R-*genes in rotation (*Me3* (DH149) the first year, *Me1* (DH330) the second year, then *Me3* (DH149) the third year), 4) the mixture of lines bearing single *R-*genes (*Me1* (DH330) or *Me3* (DH149), respectively) sown in the same plot, 5) the pyramiding of two *R-*genes (*Me3* and *Me1*) in one plant (F1 [DH149 x DH330]) and 6) the susceptible cultivar (DLL) as control. Each one scare-meter plot harboured five plants of a given modality from April-May to October, followed by five susceptible lettuces from November to February.

The experiment was carried out in a tunnel of 224 m2 (Figure 3) subdivided in 52 microplots of one square meter each naturally infested with a mixture of *Meloidogyne incognita* and *M. arenaria* as described in Material and method. Each scare-meter plot harboured five plants of a given pepper modality from April-May to October, followed by a cultivation cycle of susceptible lettuce in winter. Six cultivation modalities were compared during three years: 1) the succession of the same *R-*gene (*Me1*) introgressed in a partially resistant genetic background (DH330), 2) the succession of the same *R-*gene (*Me1*) in a F1 hybrid issued from a cross with a susceptible genotype [DH330 × DLL]), 3) the alternance of single *R-*genes in rotation (*Me3* (DH149) the first year, *Me1* (DH330) the second year, then *Me3* (DH149) the third year), 4) the mixture of lines bearing single *R-*genes (*Me1* (DH330) or *Me3* (DH149), respectively) grown in the same plot, 5) the pyramiding of two *R-*genes (*Me3* and *Me1*) in a single genotype (F1 [DH149 × DH330]) and 6) the susceptible cultivar (DLL) as control.

**Figure 4:**
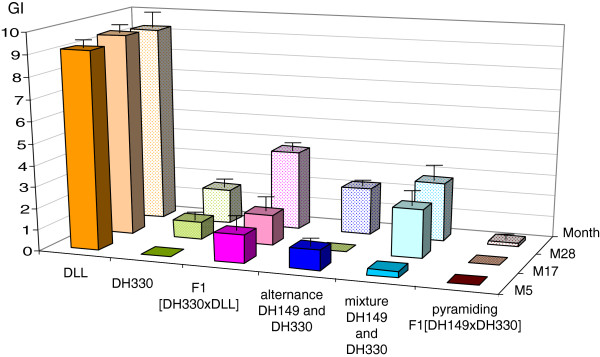
**Gall index (GI) on peppers (mean of 40 to 45 replicates ± standard error) over the 3-years field experiment.** GI checked at five (M5), 17 (M17) and 28 (M28) months. For mixture of *R-*genes, the reported value is the mean GI on DH149, no gall being observed on DH330. DLL was the susceptible cultivar as control, DH149 is homozygous resistant *Me3/Me3*, DH330 is homozygous resistant *Me1/Me1*.

The evolution of the root infestation of peppers, during the three successive years for the six modalities, respectively, is presented in Figure 4. The gall index (GI) was determined using a 0 to 10 scale as described in Material and method. As expected, the susceptible cultivar DLL, cultivated in naturally-infested plots, exhibited high infestation levels over the whole experiment (GI ranging from 9.2 to 9.4). Conversely, the five modalities that include *R*-genotypes showed a significant reduction of the number of galls on their root systems (χ^2^ = 30 to 58, ddl = 17, p = 0.05), whatever the *R*-gene(s) and the management strategy considered (i.e., alternance, mixture or pyramiding). However, differences were noticed among the five modalities. After one year of cultivation, the homozygous line DH330 did not show any gall (in monoculture or in alternation with DH149), while the level of infestation progressively increased during the second and third year (GI raised up to 1.6 the third year). The same trend was generally observed for the other modalities, the highest infestation level being observed in the case of the heterozygous F1 [DH330 × DLL] after the third year of cultivation (GI = 3.7). For the mixture, galls were only observed on DH149 (GI reached 0,29 the first year to 2,75 the third year). The only notable exception is reported for the F1 [DH149 × DH330] pyramiding *Me3* and *Me1*, which remained almost uninfested over the three years (GI ranging from 0 to 0.2). In order to evaluate the potential selection of *M. incognita* isolates virulent against *Me1* or *Me3* during the experiment, eggs recovered from *R-*peppers were hatched and the resulting second-stage juveniles (J2s) used to reinoculate the same *R-*genotype. EMs sampled on DH149 contained more than 900 eggs on average, and a virulent line was successfully reared by successive re-inoculation on DH149 peppers. After three successive re-inoculations, the mean number of eggs per EM was 866.7 ± 43.1 (18 replicates; data not shown). Few or numerous EMs were recovered from *Me1* peppers, homozygous (DH330) or heterozygous ([DH330 × DLL]), respectively, but they contained few eggs (<65 eggs per EM, data not shown). The eggs obtained the first and second year on both genotypes, and the third year on DH330, did not survive to the first re-inoculation. Nevertheless, some nematodes obtained from eggs collected the third year on heterozygous F1 ([DH330 × DLL]) survived and were reared by successive inoculations on the same genotype. After three successive re-inoculations, only 38 EMs were obtained (RR not determined, only one generation being obtained). Very few EMs were recovered from the F1 [DH149 × DH330] peppers combining *Me1* and *Me3*, and no virulent population was obtained after re-inoculation on *R*-plants (data not shown).

**Figure 5:**
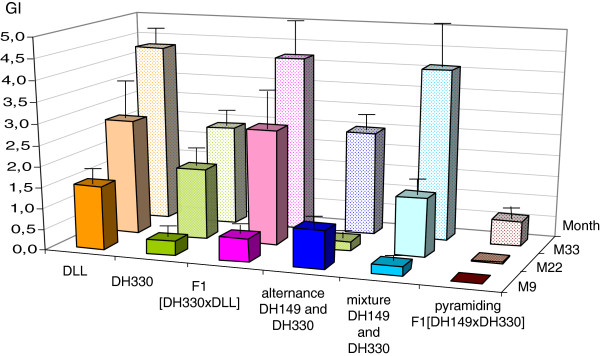
**Gall index (GI) on lettuces (mean of 40 to 45 replicates ± standard error) of the 3-years field experiment.** GI checked at nine (M9), 22 (M22) and 33 (M33) months. DLL was the susceptible cultivar as control, DH149 is homozygous resistant *Me3/Me3*, DH330 is homozygous resistant *Me1/Me1*.

Figure 5 shows the GI on susceptible lettuce each year after each pepper modality. Lettuce plants were cultivated in winter when the cycle of nematodes is slowed. So the mean GI did not exceed five on a scale from 0 to 10. Moreover, the lettuces were harvested in February the first year, when air temperature did not exceed 7°C; the second and third year, they were harvested in March with air temperatures reaching 16°C to 20°C, respectively. This could explain that the GI were higher the second and third years on the lettuces for all modalities.

After the first year of pepper cultivation, a significant reduction of GI (χ^2^ = 13 to 34, ddl = 17, p = 0.05) was observed in the lettuce cultivated after pepper *R*-genotypes compared to lettuce cultivated after the susceptible pepper DLL (mean GI = 0 after *R*-genes pyramiding to 0.9 after *R*-genes alternance compared to 1.5 after DLL). Differences were noticed among the five modalities after two additional cycles of cultivation. For three modalities, i.e., susceptible peppers, cultivation of F1 (DH330 × DLL) and mixture of resistant peppers carrying *Me1* or *Me3*, no protection effect on lettuce was observed after three cultivation cycles. Contrarily, the three other modalities, i.e., DH330, alternance, and especially pyramiding of *Me1* and *Me3* allowed protecting the lettuces during the three years: GI raised up to 4.3 in year three after DLL and only 0.6 after F1 [DH149 × DH330] peppers combining *Me1* and *Me3*.

**Figure 6:**
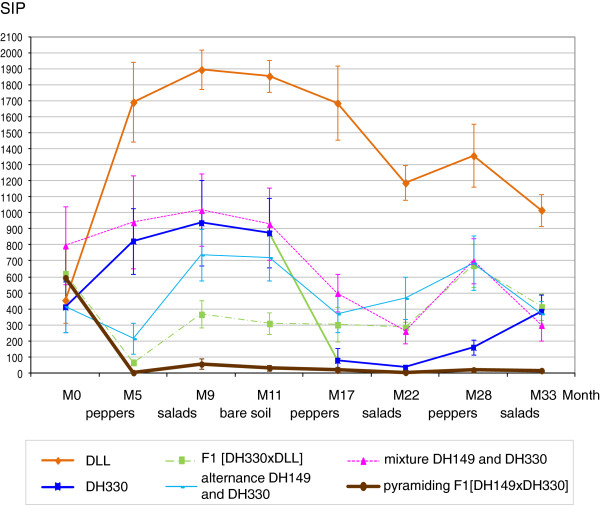
**RKN soil infection potential (SIP) of the 3-years field experiment as expressed by the number of egg-masses on susceptible tomato plants maintained six weeks in pot filled with one kg-rhizospheric soil sampled from each plot at 15 cm depth (mean of height to nine replicates ± standard error).** M = month. DLL was the susceptible cultivar as control, DH149 is homozygous resistant *Me3/Me3*, DH330 is homozygous resistant *Me1/Me1*.

These results are in agreement with those comparing the evolution of RKN soil infection potential (SIP) during the three years of experiment (Figure 6). The SIP was evaluated counting the number of EMs on susceptible tomato plants inoculated with one kg-rhizospheric soil sampled from each microplot at 15 cm depth before and after peppers or lettuces as described in Material and method. In the first year of the experiment, before planting the pepper genotypes, the SIP of the whole plot was moderate to heavy (the mean SIP for 52 microplots reached 546 ± 71 EMs per plant). The succession of susceptible plants every year (DLL in summer and lettuces in winter) greatly increased SIP in corresponding microplots (from 456 ± 140 to 1019 ± 100 EMs per plant in year 3). After two months of bare soil, no significant changes in SIP could be observed. Resistant peppers DH330, F1 [DH330 × DLL], and mixture DH330 and DH149 did not significantly reduce the SIP over three years of experimentation compared to SIP before planting the peppers. In contrast, the results highlight the beneficial effects of two management strategies of resistance: the cultivation of hybrids combining two resistance factors and alternating rotation of varieties, each carrying a different resistance. Considering the *Me3/Me1* alternance, the SIP doubled after the first year of DH149 (*Me3*) cultivation (from 412 ± 157 to 823 ± 204 EMs per plant) with the emergence of a *Me3*-virulent population. The second year, after DH330 (*Me1*) cultivation the SIP was reduced from 874 ± 218 to 78 ± 76 (i.e., 91%). However, the SIP increased again the third year after DH149 (*Me3*) cultivation. Considering the F1 [DH149 × DH330] combining *Me1* and *Me3* strongly reduced the SIP as soon as the first year (from 596 ± 179 to 2.8 ± 2.8, i.e., 99.5%), this reduction being almost complete in some microplots, when hairy root peppers were particularly developed through addition of an organic amendment and proper fertirrigation. This “trap plant” effect was maintained over the three years. The final level of reduction using this modality was 97.4% of the mean initial rate recorded in the 45 plots.

## Discussion

The laboratory and field experiments performed in this study aimed at assessing different strategies of host resistance management for their impact in three main components of the crop protection against root-knot nematodes: the ability to delay or prevent the adaptation of the pathogen to the resistance (i.e., the durability of the resistance), the efficiency in protecting the crop carrying the *R*-genes (i.e., the resistance level), and the potential in protecting the subsequent (and often susceptible) crops included in the cropping system. In the following discussion, we will compare the performances of the different strategies in addressing these three aspects.

### The durability of the resistance depends on the resistant genotype and management strategies

We showed that the choice of the *R*-gene is of crucial importance: unlike *Me3,* that was rapidly overcome after successive re-inoculations in laboratory experiments or by natural field isolates, *Me1* was either not or difficult to overcome under laboratory conditions or field conditions and only when it was weakened in a very susceptible genetic background. This validates, under natural field conditions, previous results obtained under controlled conditions [37,38] which also showed that these two genes induce distinct resistance mechanisms, the *Me3* gene inducing early cellular necrosis in the root epidermis adjacent to the juveniles whereas *Me1* induces a late hypersensitive reaction in the vascular cylinder of infected roots, thus inhibiting the development of egg-laying females [39,40]. The importance of the choice of the *R-*gene for a better durability of resistance linked to the mechanism of resistance was also pointed out in several pathosystems [7,42,43]. It showed that a few strong *R-*genes can be deployed for long periods and that results obtained in laboratory or small field experiments can adequately reflect the mechanisms acting over large geographical scales and longer time periods. Despite the higher durability of the *Me1* gene, our field experiments showed that the gall index increased in the F1 (DH330 × DLL) plots, with the potential emergence of a new *Me1*-virulent population. Previous report [41] already showed that the efficiency of *Me1* and *Me3* genes did not depend on allelic dosage (homozygous/heterozygous) but on the genetic background. The durability of this gene may have been affected by its heterozygous status in the F1 or by the susceptible genetic background of this F1 as shown in other pathosystems [8,9,44]. These studies suggested the presence of additional genes or quantitative trait loci (QTL) that may have epistatic interactions with the primary resistance determinants, or may increase the number of virulence mutations required in the pathogen genome to breakdown the resistance, as suggested by Palloix *et al.*[45]. In pepper, experiments are now underway to detect and localize such QTL, and to determine their effectiveness in protecting the major *R*-genes.

When comparing the number of EMs produced by the avirulent isolate and those produced by the virulent ones (*Me3*-virulent laboratory-selected and *Me3*-virulent natural) on the susceptible pepper DLL (Figure 2), we observed significant differences for avirulent versus *Me3*-virulent laboratory-selected (χ^2^ = 13, ddl = 2, p = 0.002) and the same tendency, but not significant, for avirulent versus *Me3*- naturally virulent (χ^2^ = 5, ddl = 2, p = 0.05). However, the difference became significant in this last case comparing the RR (number of eggs/number of juveniles inoculated, data not shown) (χ^2^ = 13, ddl = 2, p < 10–3). The present study reinforces our previous observations suggesting that a fitness cost reduces the nematode reproduction on the susceptible plants because of unnecessary virulence [37]. The virulence cost (Ch) was estimated as follows: Ch (%) = 1—RR(virulent line)/RR(avirulent line) [46]. Here, the virulence costs on susceptible pepper were estimated at 72.8% for the *Me3*-virulent laboratory-selected isolate and at 58.8% for the *Me3*-virulent natural isolate.

Alternating the two *R*-genes in rotation proved partly efficient in decreasing the nematode populations. Indeed, the nematode population selected by the *Me3* pepper the first year was strongly depressed by the *Me1* pepper the second year, but increased again with *Me3* pepper the 3rd year. This probably resulted from the variations in population size of the *Me3*-virulent nematodes. Indeed, Castagnone-Sereno *et al*. and Djian-Caporalino *et al.* showed, in different nematode populations, that the virulence gain towards *Mi-1*(tomato), *Me1* or *Me3* in pepper was specific of the targeted *R*-gene [37,38]. The alternating strategy proved partly efficient in decreasing temporarily the pre-existing *Me3*-virulent population. Because of the absence of cross-virulence, alternating with *Me3* peppers is reciprocally expected to prevent the emergence of new *Me1*-virulent variants as observed in the continuous deployment of *Me1* in the F1 hybrid.

The mixture strategy (cultivation of *Me1* and *Me3* peppers in the same plots) did not provide a significant protection of *Me3*-peppers since the *Me3*-virulent fraction of the nematode population increased continuously over the three years with increased damages on *Me3*-peppers. The mixture strategy may have decreased the level of epidemics due to the reduced density of susceptible plants as advocated by Pink [47]. However we got no evidence that it delayed the increase in frequency of virulent nematodes, despite the very limited dispersion ability and absence of sexual reproduction of RKNs which are expected to favour resistance durability [2].

The pyramiding of the two *R*-genes proved to be the only management practice which totally suppressed the emergence of virulent isolates, both in laboratory with high inoculum density experiments and in the 3-year field experiment. In theory, pyramiding into a single cultivar several *R*-genes that have the same spectrum of action but that differ in their mechanisms should provide a more durable resistance, since mutational events at several avirulent loci would be required simultaneously to produce a new virulent pathotype [10,48]. The pre-existence of *Me3*-virulent nematodes in the field may endanger the pyramiding strategy, facilitating the further emergence of both *Me3* and *Me1*-virulent nematodes. However, no multivirulent genotype emerged, that probably resulted from the extremely low nematode population in these plots after one year of F1 [DH149 × DH330] peppers cultivation, preventing the emergence and selection of such genotypes.

Comparing the management strategies for their ability to protect the resistant cultivars from virulence emergence, provided contrasted results in our experiment. The continuous use of a single *R*-gene, (although *Me1* was previously considered for its potential high durability) proved a risky strategy with the potential emergence of virulence after three years, particularly when the *R*-gene was introgressed in a susceptible background. The mixture of *R*-lines proved not efficient, due to the increase in frequency of a pre-existing virulent nematode genotype. Alternating two *R*-genes that differ in their mechanisms seemed partly efficient and offered the possibility of ‘recycling’ broken *R*-genes due to the strict specificity of virulence. Finally, pyramiding two major *R*-genes that differ in their mechanisms into a single cultivar seemed the most secure and durable strategy.

### Efficiency of the management strategies in protecting the successive drops in the rotation

These management strategies were also compared for their ability to minimize damage in the targeted crop (pepper) and to decrease the soil infectious potential, thus securing crop rotation including further susceptible hosts. The continuous cultivation of *Me1* peppers provided different results depending on the carrier genotype: the DH line 330 proved efficient in decreasing the damage in pepper and in lettuce (low GI compare to the susceptible DLL pepper). This was related with a low to intermediate SIP all along the three cultivation cycles. Conversely, with the F1 (DH330 × DLL), the GI in pepper and lettuce increased progressively to intermediate (pepper) or very high (lettuce) levels, and the SIP remained rather high. This is related to the increase in EM numbers observed in the F1 hybrid compared to the resistant parent DH330 in laboratory experiments. Introgressing the *R*-gene in susceptible cultivars with heterozygous status decreases the efficiency of the resistance and results in an increase in the reproduction and maintenance of the pathogen population. This was already known in other pathosystems such as tomato-*M. incognita*, cotton-*M. incognita*, and rice-*Xanthomonas oryzae*[49-51]. Recent results indicated that this effect is due to the genetic background rather than the heterozygocity of the *R*-gene [41]. It clearly confirmed that the way of breeding resistant cultivars can affect the efficiency of the RKN *R-*genes in addition to their durability, as previously mentioned.

In presence of a *Me3*-virulent population, alternating with another *R*-gene like *Me1* was efficient to cut down the nematode population (Figure 6) and to protect the susceptible crops in the rotation (Figure 5). It allowed using again the broken *R*-gene *Me3* in the following cycle of cultivation and provided a partial protection of the susceptible lettuces despite the significant increase of the SIP after *Me3 R*-peppers. This indicated that *Me1* will need to be used again to decrease the *Me3*-virulent population that developed in the previous cycle and maintain the SIP at acceptable level.

Mixture of DH330 and DH149 did not significantly reduce the SIP over three years of experimentation, except the first year when the roots were highly developed. Implementing a root growth stimulation when using *R*-plants could so increase the “trap plant” effect and thus decrease the amount of pathogens in the soil. A multiline protocol was tested to control rice blast (caused by *Magnaporthe grisea*) in Yunnan province, China, with striking success [22]. In the present experiment, the SIP value did not increase as much as observed with a succession of susceptible crops (DLL and lettuce), however it was maintained, probably due to the selection of *Me3*-virulent nematodes. This reduced the mean GI in pepper, but resulted in a high GI on lettuces after three years, reaching that obtained after DLL susceptible peppers. In this situation, the mixing strategy proved not efficient to protect the susceptible crop on the long term.

The pyramiding strategy proved highly efficient in protecting the pepper crop, as expected from laboratory experiments, but also the following susceptible crop all along the three years. Indeed, the SIP fell down as soon as the first cultivation of peppers combining the two *R*-genes and never increased again. Peppers are not known for any nematicide activity, but very probably act as traps. The use of trap plants is a nematode management technique that has been tested periodically since the late 1800’s (e.g., [52,53]). Nematicidal plants used include *Arachis hypogeae* (peanut), *Cucumis metuliferus* (a wild melon) or *Solanum sisymbriifolium*[54-56] but are difficult to introduce in vegetable crop rotations. Susceptible hosts (carrot, radish, cucumber) have also been used to trap RKN juveniles, but have to be destroyed before the completion of the life cycle of the nematode [57,58]. *R*-plants are more efficient trap crops as nematodes are killed in the roots, avoiding the destruction of the plants before the end of the nematode life cycle. *R*-peppers, specially the F1 [DH149 × DH330] peppers combining *Me1* and *Me3,* provide a new trap crop in intercropping and integrated pest management, and for crop diversification in vegetable cropping systems.

To conclude on the strategies that minimize damage in the targeted crop and decrease the soil infection potential, securing crop rotation including susceptible hosts, the sequential use of a single *R*-gene introgressed in a susceptible background proved inefficient and the mixture of *R*-lines also lost efficiency as soon as one of the *R-*gene was broken down. Alternating two *R*-genes that differ in their mechanisms was partly efficient in maintaining a low inoculum level in the long term, but pyramiding two major *R*-genes into a single cultivar outcompeted the other strategies, providing a high protection level of the resistant cultivar (no multivirulent emerged, neither in laboratory with high inoculum pressure, nor in the 3-yrs field experiments) and a causing a sharp and stable drop of the soil infectious potential. Moreover, the availability of molecular markers closely linked to each of the *Me R*-genes [35,59] makes the identification of digenic genotypes possible and will help breeders to construct novel resistant pyramid genotypes.

## Conclusion

Looking at three components of crop protection –durability of resistance, efficiency of resistance, and sustainability of rotating cultivation- provided the same hierarchy of management strategies with Pyramiding > Alternating > Mixture > Sequential use of a single *R*-gene introgressed in a susceptible background. Looking to the adaptation of xenobiotic to drugs and pesticides, a very similar hierarchic efficiency of strategies was observed by the Resistance to Xenobiotics Consortium [27]. Based on literature review, they showed that the combination of molecules (equivalent to the pyramiding) outcompeted the other strategies (sequential use, mixing or alternating) in delaying the emergence of resistance to drugs and pesticides. The superiority of molecules combination over the other strategies appeared to be robust particularly when resistance to each drug in the combination was controlled by independent loci (no cross-resistance), leading to “multiple intragenerational killing” of the pathogens at the individual level. *Me1* and *Me3* are two distinct *R*-genes with different modes of action [39,40] and no cross-virulence effects [36]. Moreover, pyramiding appeared very promising as RKN “traps” plants, reducing up to 90% the infestation rate of the soil, protecting the winter susceptible crops. Decreasing the amount of pathogens in the soil may also increase the durability of *R*-genes because the appearance and early increase in the frequency of virulence alleles in the pathogen population depends on the balance between mutation rates and population size [27]. When pyramiding remains difficult for breeders, alternating may offer the possibility of ‘recycling’ broken *R*-genes provided that virulence is specific. Results of this study are expected to suggest rules for breeders and farmers for the sustainable management of disease resistance.

## Methods

### Plant material

The five pepper (*Capsicum annuum*) genotypes used in this work are inbred lines with differential resistances to RKNs. Doux Long des Landes (DLL) is a susceptible cultivar. The two resistant doubled-haploid (DH) lines, DH149 and DH330 produced through *in vitro* androgenesis were previously described [33]; they are homozygous for the *Me3* and *Me1* genes, respectively [39]. The selection of virulent variants against the *Me3* gene was achieved through strong inoculation pressure by avirulent *M. incognita* isolates [36,38]. Under laboratory conditions, *Me1* prevents the emergence of *Me1*-virulent nematode genotypes, despite the implementation of drastic levels of inoculum [37]. Two F1 hybrid were also used, one carrying *Me1* in its heterozygous state in the DLL susceptible genetic background (F1 [DH330 × DLL]), and one combining the two *R-*genes *Me3* and *Me1* (F1 [DH149 × DH330]). All the lines were produced independently in insect-proof cages to eliminate outcrossing. Pepper seedlings were grown individually in 100 mL pots containing steam-sterilized sandy soil covered by a one cm layer of loam in climatic chambers maintained at 24°C (±2°C) with a 12-h light cycle and a relative humidity of 60–70%.

### Climate controlled room experiments

The experiments were conducted in a climatic chamber maintained at 24°C (±2°C) with a 14-h light cycle and a relative humidity of 60–70%. Three *M. incognita* isolates were used. Morelos is a *Me1-* and *Me3*-avirulent isolate from the collection maintained at INRA Sophia Antipolis on susceptible tomatoes cultivar Saint Pierre. From the Morelos avirulent isolate, a *Me3*-virulent laboratory-selected line was obtained and reared by successive re-inoculation on the *Me3*-pepper DH149 line for more than 25 generations, starting from the progeny of one single female, according to the procedure of Jarquin-Barberena *et al.*[60]. The *Me3*-virulent natural isolate was collected on resistant peppers carrying *Me3* in the experimental field of CREAT, La Baronne, France. It was isolated after one single year of use of *Me3* in the field (approximately three nematode generations). Prior to multiplication, each isolate was specifically identified according to its isoesterase electrophoretic pattern [61] and/or by sequence characterised amplified region based PCR assays (SCAR-PCR) [62].

Hatched second-stage juveniles (J2s) were obtained in a mist chamber from previously inoculated roots. Nematodes were collected in water every 48 hours and used immediately to inoculate the plants. The five pepper genotypes were compared. For each nematode isolate, six to seven-week-old plants (4–6 true leaves) produced in climatic chambers were inoculated with a water suspension of 500 J2s. To compare the reproduction rate of the nematodes, fifteen to twenty plants were analyzed for each cultivar × nematode combination tested. To evaluate the ability of the nematodes to overcome the pepper *R-*genes in different genetic backgrounds, 70 to 100 plants of each *R*-line (17 plants for the susceptible control DLL) were inoculated with 5000 J2s of avirulent *M. incognita* Morelos.

### Design of the 3-yrs field experiment

The experiment was carried out in a plastic tunnel belonging to the Chamber of Agriculture of Alpes-Maritimes (technical institute) in La Gaude (Sud East of France). The tunnel was 224 m2 (28 m × 8 m). The soil had a pH of 8.2 with 46.68% of sand, 27.99% of loam, and 25.33% of clay. Total CaCO3 was 171 g/kg. The soil temperature in the tunnel varied from 15°C in winter (December to April) to 25°C in summer (June to September) at 15 cm depth (Mediterranean climate). During the whole experiment, the tunnel received no phytosanitary treatment. It was subdivided in 52 microplots of one square meter each, separated by one meter of bare soil between each plot (Figure 3). Before starting the experiment, nematode-susceptible tomatoes were cultivated for three consecutive years in non disinfected soil which was naturally infested with a mixture of *Meloidogyne incognita* (detected in all the 52 plots) and *M. arenaria* (detected in 12 plots). Nematodes sampled from the roots (a mean of 10 females per plot) were specifically identified according to their isoesterase electrophoretic pattern [60] and/or by SCAR-PCR [62]. The experiment was performed on 4 rows (one meter apart) with two lines of fertirrigation drips (16 mm diameter tubes with 10 holes/m^2^ providing two L/hour) by rank and the establishment of a non-degradable plastic mulch to prevent contamination between plots. Seven to eight-week-old plants (8–10 true leaves) produced in climatic chambers were transplanted in the plots. The first year, the experiment received an organic amendment before the establishment of the plastic mulch. The third year, the experiment was only irrigated but not amended by the grower.

Six cultivation modalities were compared during three successive years: 1) the succession of the same *R*-gene (*Me1*) introgressed in a partially resistant genetic background (DH330), 2) the succession of the same *R*-gene (*Me1*) in a F1 hybrid issued from a cross with a susceptible genotype [DH330 × DLL]), 3) the alternance of single *R*-genes in rotation (*Me3* (DH149) the first year, *Me1* (DH330) the second year, then *Me3* (DH149) the third year), 4) the mixture of lines bearing single *R*-genes (*Me1* (DH330) or *Me3* (DH149), respectively) grown in the same plot, 5) the pyramiding of two *R*-genes (*Me3* and *Me1*) in a single genotype (F1 [DH149 × DH330]) and 6) the susceptible cultivar DLL as control.

Each scare-meter plot harboured five plants of a given modality from April-May to October, followed by a cultivation cycle of susceptible lettuce (*Lactuca sativa* cultivar Dedale-batavia), from November to February. Repeats of height to nine plots and 40 to 45 plants per genotype were tested, respectively (Figure 3).

### Infestation parameters

For climate controlled room experiments, the plants were harvested six to seven weeks after inoculation (i.e., a duration that allowed completion of the nematode life cycle). Roots were carefully washed individually with tap-water, and stained for 10 minutes in a cold aqueous solution of eosin yellow (0.1 g/liter water), to specifically stain egg-masses (EMs) in red [63]. The roots were then rinsed and examined under a magnifying glass and the number of EMs counted for each plant.

For field experiments, several infestation parameters were analysed along the three years: the gall index (GI), the RKN soil infection potential (SIP), and the reproduction rate (RR) of virulent RKNs, if they were detected.

GI was determined for the roots of each pepper or lettuce plant using a 0 to 10 scale [64]. The number of infected plants per genotype tested was also recorded.

To determine SIP, five replicates of one kg-rhizospheric soil were sampled from each plot at 15 cm depth before and after peppers or lettuces. Two-month-old susceptible tomato plants (cultivar Saint Pierre) were transplanted in pots filled with these soil samples and maintained in greenhouses. After six weeks, the number of EMs on the tomato plants was evaluated as previously described.

To determine RR of potentially virulent nematodes, EMs detected on a resistant pepper were picked and inoculated on a 2-month-old resistant pepper carrying the same *R-*genes(s) and maintained in a climatic chamber (24°C ± 2°C, 14-h photoperiod). After six weeks, the roots were carefully washed with tap-water and examined under a magnifying glass to detect EMs. If EMs were detected, they were reared by successive re-inoculations on 2-month-old resistant peppers carrying the same *R-*genes(s) according to the procedure of Jarquin-Barberena *et al*. [60]. After two generations, 10 EMs were picked up and the mean number of eggs per EM (i.e., the number of eggs produced by one female) was evaluated.

### Statistical analysis

The effect of treatments or pepper genotypes on the parameters (SIP, RI, RR) was tested with a Kruskal-Wallis test. The mean values of treatments or genotypes were compared using the Wilcoxon-Mann–Whitney unilateral test. Bonferroni correction was applied to use a significance level at α = 0.05. Analyses were performed using the free software R (http://www.r-project.org/).

## Abbreviations

Ch: Virulence cost; DH: Doubled-haploid; DLL: Doux Long des Landes; EM: Egg-mass; GI: Gall index; HR: Hypersensitive reaction; J2s: Second-stage juveniles; M. incognita: Meloidogyne incognita; M: Months; QTL: Quantitative trait loci; R-gene: Resistance gene; R-genotype: Resistant genotype; RKNs: Root-knot nematodes; R-plant: Resistant plant; RR: Reproductive rate; SCAR-PCR: Sequence characterised amplified region based PCR assays; SIP: Soil infection potential.

## Competing interests

The authors declare that they have no competing interests.

## Authors’ contributions

CDC conceived of the study, coordinated and carried out the experimentations, and drafted the manuscript. PCS and TM participated in the design of the study and in carrying out the experimentations, and helped to draft the manuscript. AP provided the pepper accessions and progenies, participated in the design of the study and in carrying out the experimentations, and helped to draft the manuscript. PA coordinates researches of the Plant-Nematode Interaction group and helped to conceive the research program and this study. AF, NM, AB, AMSP, SR, RL, CT participated in carrying out the experimentations. All authors read and approved the final manuscript.
